# Functional Effects of Bilateral Dorsolateral Prefrontal Cortex Modulation During Sequential Decision-Making: A Functional Near-Infrared Spectroscopy Study With Offline Transcranial Direct Current Stimulation

**DOI:** 10.3389/fnhum.2020.605190

**Published:** 2021-02-03

**Authors:** Iryna Schommartz, Annika Dix, Susanne Passow, Shu-Chen Li

**Affiliations:** ^1^Chair of Lifespan Developmental Neuroscience, Faculty of Psychology, Technische Universität Dresden, Dresden, Germany; ^2^Department of Developmental Psychology, Institute of Psychology, Goethe University Frankfurt, Frankfurt, Germany; ^3^Centre for Tactile Internet With Human-in-the-Loop, Technische Universität Dresden, Dresden, Germany

**Keywords:** bilateral tDCS, fNIRS, DLPFC, sequential decision-making, three-stage Markov task

## Abstract

The ability to learn sequential contingencies of actions for predicting future outcomes is indispensable for flexible behavior in many daily decision-making contexts. It remains open whether such ability may be enhanced by transcranial direct current stimulation (tDCS). The present study combined tDCS with functional near-infrared spectroscopy (fNIRS) to investigate potential tDCS-induced effects on sequential decision-making and the neural mechanisms underlying such modulations. Offline tDCS and sham stimulation were applied over the left and right dorsolateral prefrontal cortex (dlPFC) in young male adults (*N* = 29, mean age = 23.4 years, SD = 3.2) in a double-blind between-subject design using a three-state Markov decision task. The results showed (i) an enhanced dlPFC hemodynamic response during the acquisition of sequential state transitions that is consistent with the findings from a previous functional magnetic resonance imaging (fMRI) study; (ii) a tDCS-induced increase of the hemodynamic response in the dlPFC, but without accompanying performance-enhancing effects at the behavioral level; and (iii) a greater tDCS-induced upregulation of hemodynamic responses in the delayed reward condition that seems to be associated with faster decision speed. Taken together, these findings provide empirical evidence for fNIRS as a suitable method for investigating hemodynamic correlates of sequential decision-making as well as functional brain correlates underlying tDCS-induced modulation. Future research with larger sample sizes for carrying out subgroup analysis is necessary in order to decipher interindividual differences in tDCS-induced effects on sequential decision-making process at the behavioral and brain levels.

## Introduction

Everyday decision-making often involves considerations about short- or long-term goals that entail learning from immediate or delayed action–outcome associations for updating one's behavior. Reaching long-term goals often presupposes transitioning between different decision states, involving complex value-based learning or adaptive assignment of values to sequential actions based on the outcome (Puterman, 1990; Beroggi, 2013; Preuschoff et al., 2013). These abilities and their underlying neural mechanisms have previously been investigated (see Badre and D'Esposito, 2009 for a review; Tanaka et al., 2004; Smittenaar et al., 2013; Eppinger et al., 2015; Wittkuhn et al., 2018). However, possible enhancements of sequential decision-making by non-invasive brain stimulation techniques, such as transcranial direct current stimulation (tDCS), are still not well-understood. In particular, to date, it is not known whether tDCS may modulate brain hemodynamic responses during complex sequential decision-making that requires learning about state-dependent action–outcome associations and whether tDCS-induced effects at the brain level may directly contribute to performance enhancement.

Previous research has investigated the underlying neural correlates of individuals' sequential decision-making performance by using different variants of a deterministic three-stage Markov task (Tanaka et al., 2004; Eppinger et al., 2015; Wittkuhn et al., 2018). This task includes two conditions. In the immediate reward condition, one choice option is associated with a small positive reward and the other option with a small negative reward in all three states of the decision sequence. In comparison, in the delayed reward condition, one choice option is accompanied by two small positive rewards in the first and second states and a big loss in the third state. Conversely, the other choice option is accompanied by small losses in the first two states and a big reward in the third state. Structured as such, the optimal strategy to obtain a net positive and a higher amount of overall reward in such a three-stage Markov decision task is to make decisions in favor of small positive rewards in the immediate reward condition, whereas one needs to accept small losses in the first two states in the delayed reward condition, in order to gain a big reward in the third state. Applying functional magnetic resonance imaging (fMRI) to examine the brain functional correlates of sequential decision-making in this task revealed a network of frontal regions including the prefrontal cortex (PFC). Specifically, the ventrolateral PFC, the lateral orbitofrontal cortex, the insula, the left rostrolateral PFC, the right medial PFC, and the dorsolateral PFC (dlPFC) are strongly involved in the ability to extract sequential state transition structures while learning to predict future rewards (Tanaka et al., 2004; Eppinger et al., 2015). Furthermore, the involvement of the aforementioned prefrontal regions differs between younger and older adults. Eppinger et al. (2015) showed that impaired learning of sequential action–outcome contingencies contributing to older adults' lower performance in learning from long-term outcomes was accompanied by significant under-recruitments of the right dlPFC, left rostrolateral PFC, and right medial PFC. On the other hand, higher blood-oxygen-level-dependent activity in the left dlPFC in younger adults was associated with optimal choices in the crucial transition states in the delayed reward condition during the course of learning.

Evidence for the causal involvement of the right dlPFC in sequential decision-making was previously shown by applying inhibitory theta-burst transcranial magnetic stimulation over the right but not the left dlPFC. Theta-burst transcranial magnetic stimulation impaired the complex flexible model-based decision-making in a two-state Markov decision task in younger adults (Smittenaar et al., 2013). In addition, further causal evidence has also been obtained in an earlier work by our group (Wittkuhn et al., 2018), which applied an inhibitory 1-Hz repetitive transcranial magnetic stimulation protocol over the left dlPFC and found significantly impaired decision performance that required the learning of sequential transitions (Wittkuhn et al., 2018).

Non-invasive brain stimulation methods can be used, however, also to induce performance-enhancing modulation of different cognitive functions (Wagner et al., 2007; Valero-Cabr et al., 2011). Accumulating evidence shows that tDCS as a promising tool of neuromodulatory interventions (Kadosh, 2014) applied over the dlPFC could enhance performance in different kinds of decision-making (see Jacobson et al., 2012; Coffman et al., 2014; Tremblay et al., 2014; Thams et al., 2020a for an overview). Other studies showed that tDCS may enhance neuroplasticity and could lead to corresponding long-term changes (Fregni et al., 2005; Fecteau et al., 2007a; Dockery et al., 2009; Thams et al., 2020a). For instance, it has been shown that bilateral right anodal and left cathodal (RALC) tDCS increased response confidence in a risky decision-making task (Minati et al., 2012), decreased risk-taking behavior in an ambiguous adaptive decision-making task (Fecteau et al., 2007a,b), enhanced reflective judgment and decision-making in cognitive reflection and representativeness heuristic tasks (Edgcumbe et al., 2019), and resulted in more advantageous decision-making in the Iowa Gambling Task as well as in the Wisconsin Card Sorting Task (Soyata et al., 2019). Critically, Fecteau et al. (2007b) reported that, compared with bilateral tDCS, unilateral anodal tDCS over the left or right dlPFC was not sufficient to modify risk-taking behavior significantly in a sample of young adults performing the Balloon Analog Risk Task. Based on these previous findings and taking into the consideration that there are only a few findings on tDCS-induced neural and behavioral modulations of sequential decision-making (Smittenaar et al., 2014), bilateral RALC tDCS over the dlPFC seems to be a promising candidate tDCS setup to enhance sequential decision-making. Therefore, in the present study, we applied bilateral RALC tDCS over the dlPFC while younger adults performed a three-state Markov decision task.

Thus far, functional brain mechanisms underlying tDCS-induced enhancement of sequential decision-making are not yet well-understood. To fill this gap, we applied functional near-infrared spectroscopy (fNIRS), since it has been successfully used to study the neural activity of the dlPFC during different kinds of cognitive control and decision-making tasks (Cazzell et al., 2012; Bembich et al., 2014; Holper et al., 2014). As a non-invasive optical imaging method, fNIRS allows making inference about changes in neural activity by measuring local brain tissue oxygenation (Orihuela-Espina et al., 2010) through tracking changes in the main variants of hemoglobin: oxy-hemoglobin (O_2_Hb) and deoxyhemoglobin (HHb) (Delpy and Cope, 1997; Villringer and Chance, 1997; Berg et al., 2002; Perrey, 2008; Tachtsidis and Scholkmann, 2016). In comparison with other neuroimaging methods, the application of fNIRS has several advantages: for instance, it is superior to electroencephalography in localizing neural signals and is more robust against movement artifacts than fMRI. Furthermore, fNIRS is a low-cost, ecologically valid, and portable method for capturing hemodynamic changes as functional brain correlates of behavior and action (Gu et al., 2017).

In the research on decision-making, Bembich et al. (2014) examined the activation of the dlPFC while participants performed high- and low-risk choices in the Iowa Gambling task. Results from this study showed that fNIRS reliably captured O_2_Hb variations in bilateral dlPFC depending on task conditions, with increased O_2_Hb concentration changes during high-risk choices. Assessing the activity in dlPFC in a dynamic risky decision-making task, Holper et al. (2014) showed an increase in total hemoglobin (tHb), i.e., the sum of the O_2_Hb and HHb (Villringer and Chance, 1997), in response to positive outcomes (or gains) in bilateral dlPFC as well as a positive correlation between dlPFC activation and overall performance level. dlPFC activity had also been observed during moral and economic decision-making in the Ultimatum Game, which showed a reduction in HHb concentration changes during the economic condition of the task in female subjects, reflecting higher dlPFC recruitment (Vanutelli et al., 2020). Similarly, an increase in O_2_Hb and a decrease in HHb in the dlPFC were observed during moral decision-making following fair offers in the modified Ultimatum Game (Balconi and Fronda, 2020). The fNIRS has also been applied to investigate hemodynamic concentration changes in the dlPFC when individuals performed the Balloon Analog Risk Task. The findings showed a stronger O_2_Hb and HHb signal in bilateral dlPFC during active in comparison with passive decision-making (Cazzell et al., 2012), replicating previous fMRI findings (Rao et al., 2008) and showing fNIRS' ability to capture decision-making-related hemodynamic responses in the dlPFC.

Furthermore, fNIRS can be suitably combined with tDCS to investigate neural correlates of stimulation-induced modulation effects on cortical responses (Lloyd-Fox et al., 2010; Dutta et al., 2015; McKendrick et al., 2015). For instance, in a proof-of-concept study using high-definition tDCS, fNIRS successfully captured high-definition tDCS-induced hemodynamic changes, showing that anodal high-definition tDCS over the left motor cortex yielded significantly higher O_2_Hb concentration in the left motor cortex in comparison with sham stimulation (Muthalib et al., 2018). In another study that measured PFC activity during a working memory task, unilateral anodal tDCS over the left dlPFC led to a significant increase of O_2_Hb levels along with improved task performance (Jones et al., 2015). Extending these findings, Di Rosa et al. (2019) showed an increase in O_2_Hb concentration changes after anodal tDCS over the left PFC in older healthy participants while performing a reward-based working memory task. This tDCS-induced elevated hemodynamic activity was accompanied by a significant improvement of working memory performance (Di Rosa et al., 2019). Furthermore, using a high-definition unilateral tDCS over the right ventrolateral PFC, McKendrick et al. (2020) reported augmented spatial working memory performance accompanied by changes in O_2_Hb and HHb in the dlPFC and left ventral medial PFC. Taken together, studies that combined tDCS with fNIRS have shed new light on the underlying neural correlates of tDCS-induced neurocognitive enhancement.

In light of the above previously observed tDCS-induced effects on frontal regions relevant for working memory and decision-making, in this study, we aimed to investigate whether learning sequential contingencies of actions that are associated with future outcomes may also be enhanced by tDCS, since this ability is indispensable for daily decision-making contexts and was shown to deteriorate with advancing age (Eppinger et al., 2015). Based on the empirical evidence that pointed out dlPFC as a candidate region to be strongly involved in the ability to learn state-dependent action–outcome associations in younger participants while under-recruited and associated with performance deficits in older participants (Smittenaar et al., 2013; Eppinger et al., 2015; Wittkuhn et al., 2018), we defined the dlPFC to be the target region for tDCS modulation. Since little is known about neuromodulatory effects of offline tDCS on sequential decision-making and its possible contribution to performance enhancement, we aimed also to shed light on these questions by applying fNIRS to examine tDCS-induced effects on hemodynamic changes at the brain level. Thus, we combined offline tDCS with fNIRS assessing brain hemodynamic responses in a double-blind between-subject design (sham stimulation vs. tDCS) in this study. Based on prior work (Rao et al., 2008; Sato et al., 2013; Vassena et al., 2019) and as a proof-of-concept, after sham stimulation, we expected fNIRS to capture the differences in hemodynamic response between the immediate and delayed reward condition in a three-state Markov decision task similar to an earlier fMRI study (Eppinger et al., 2015). Further, since the dlPFC is not critically involved in the learning of action–outcome associations in the immediate reward condition in healthy young adults (Eppinger et al., 2015; Wittkuhn et al., 2018), higher recruitment of the dlPFC, in this case, may reflect inefficient processing that could be counterproductive for task performance (Eppinger et al., 2015; Wittkuhn et al., 2018). Therefore, the upregulation of hemodynamic response in the delayed compared with the immediate reward condition was hypothesized to be positively associated with performance in the delayed reward condition. Based on previous findings showing the effectiveness of the bilateral RALC tDCS over the dlPFC in prefrontal-based decision-making tasks (Fecteau et al., 2007a,b; Soyata et al., 2019), we expected performance-enhancing tDCS effects after tDCS compared with sham stimulation. In addition, based on previous findings indicating task-related tDCS-induced modulation of the hemodynamic response in the left dlPFC during a working memory task (Jones et al., 2015; Di Rosa et al., 2019), we hypothesized to see a higher neural response after tDCS than after sham stimulation measured by fNIRS.

## Materials and Methods

### Participants

Thirty-one young male participants were recruited to participate in the study through advertisements on the campus of Technische Universität Dresden. The focus on male participants was based on previous findings on altered cortical excitability in females due to ovarian hormonal changes during the menstrual cycle (Dietrich et al., 2001; Inghilleri et al., 2004; Hausmann et al., 2006; Harden, 2014; Zoghi et al., 2015). All participants had normal or corrected to normal vision, were right-handed regarding the assessment using the Edinburgh Handedness Inventory (Oldfield, 1971), and were screened for tDCS eligibility (e.g., no metal implants, no history of neurological or psychiatric disease, non-smokers; Fertonani et al., 2015; Woods et al., 2016). However, two participants were excluded due to incomplete task execution (*n* = 1) or technical problems during the data acquisition (*n* = 1). Thus, the remaining 29 healthy male participants constituted the final sample size [mean age: 23.4 years, standard deviation (SD) = 3.02, age range: 20–30 years]. Participants were randomly assigned to one of two stimulation conditions: 15 participants received tDCS, whereas the other 14 participants received sham stimulation.

An *a priori* power calculation was based on three identified studies addressing hemodynamic correlates of tDCS-induced effects over the dlPFC on relevant cognitive functions (e.g., working memory and decision-making) assessed by fNIRS (Jones et al., 2015; Choe et al., 2016; Herrmann et al., 2017). The generic effect size (Cohen, 1973; Lakens, 2013) of 0.47 (*f* = 0.47) was calculated based on the only reported effect size [(η^2^); η^2^ = 0.18 [*F*_(1, 21)_ = 4.45, *p* = 0.04; Jones et al., 2015]], applying G^*^Power 3.1 (Faul et al., 2009). The *a priori* power analysis was conducted for a between-subject design with a significance level of α = 0.05, statistical power (1 – β = 0.80; Cohen, 1988), and a correlation between the repeated measures *r* = 0.4, and the two groups (tDCS, sham stimulation) revealed a sample size of *N* = 28. Thus, the sample size of this study (*n* = 29; sham-stimulation group: *n* = 14; tDCS group: *n* = 15) has sufficient power to detect mean tDCS-induced hemodynamic effects reported in previous findings.

All participants gave written informed consent prior to participation. The study was approved by the Ethics Committee of the Technische Universität Dresden (EK 469112015). The participants were compensated for their participation and, in addition, received the win they earned during the three-state Markov decision task (a total average of about 10 euros).

### Assessment of Demographic, Cognitive, and Stimulation-Related Covariates

In addition to the experimental paradigm, individual differences in the motivational system (e.g., behavioral inhibition or sensitivity to punishment and behavioral activation or sensitivity to reward) were measured (BIS/BAS scales; Strobel et al., 2001). Furthermore, the motivational trait “need for cognition” that reflects how much the individual enjoys to engage in cognitively demanding activities was assessed as well (Need for Cognition Scale; Bless et al., 1991). Several psychometric tests assessing basic cognitive abilities for the estimation of between-group comparability were completed as well: (1) Wiener Matrizen Test 2 (WMT-2; Formann et al., 2011) to assess logical reasoning; (2) Identical Pictures Test (Lindenberger and Baltes, 1997) to assess perceptual speed; and (3) Spot-the-Word Test (Baddeley et al., 1993) to assess verbal knowledge. Moreover, participants filled in the German version of Positive and Negative Affect Schedule (PANAS; Krohne et al., 1996) measuring interindividual differences in the affect state before and after the experimental session. Lastly, the tDCS Adverse Effects Questionnaire (Brunoni et al., 2011) was used to assess tDCS-associated adverse effects.

Control analysis comparing the subsamples of participants based on their random appointment to either tDCS or sham-stimulation group was conducted using analysis of variance (ANOVA) on psychometric measures (see results summarized in Table 1). Subgroups of participants (tDCS group and sham-stimulation group) did not differ regarding demographic covariates, motivational traits, need for cognition trait, basic cognitive abilities (e.g., logical reasoning, perceptual speed, verbal knowledge) as well as in affect state, and tDCS adverse effects (all *p* values > 0.23).

**Table 1:** Sample characteristics by treatment group (active bilateral transcranial direct current stimulation (tDCS) compared with sham stimulation) showing comparability between groups.

	**tDCS**	**Sham stimulation**	**Group effect**	
	**(*n* = 15)**	**(*n* = 14)**		
	**Mean (SD)**	**Mean (SD)**	***p*-value**	**η^2^**
**Demographic and psychometric measures**				
Age	24.00 (3.12)	22.79 (2.91)	0.29	0.04
Years of education	16.33 (2.41)	15.71 (2.43)	0.50	0.02
Need for cognition	81.33 (14.62)	80.71 (12.68)	0.90	0.001
BAS drive	11.27 (2.91)	10.21 (2.19)	0.28	0.04
BAS fun	11.60 (2.67)	11.00 (2.48)	0.54	0.01
BAS reward	16.00 (3.17)	14.00 (3.35)	0.44	0.02
BIS	18.60 (3.85)	18.64 (4.03)	0.98	0.000
**Cognitive measures**				
WMT-2 (correct in %)	85.55 (12.36)	82.14 (14.96)	0.51	0.02
Identical pictures hit	33.47 (4.21)	33.71 (3.93)	0.87	0.001
Identical pictures RT	2,073.11 (277.97)	2,059.11 (232.63)	0.89	0.001
Spot a word hit	19.47 (5.25)	20.50 (4.69)	0.58	0.01
Spot a word RT	5,041.45 (1,350.05)	5,362.00 (2,606.00)	0.68	0.007
**Stimulation-related measures**				
PANAS positive (pre)	29.87 (3.42)	31.14 (4.69)	0.41	0.03
PANAS positive (post)	23.73 (4.76)	23.14 (6.11)	0.77	0.003
PANAS negative (pre)	12.93 (2.19)	13.5 (2.71)	0.54	0.01
PANAS negative (post)	12.57 (3.18)	13.00 (4.42)	0.77	0.003
tDCS side effects	7.80 (4.78)	5.79 (3.12)	0.19	0.06
Impedance session	3.67 (0.68)	3.70 (1.24)	0.91	0.000

*NFC, need for cognition; BIS/BAS, behavioral inhibition system/behavioral activation system; WMT-2, Wiener Matrizen test; SD, standard deviation*.

* *p < 0.05*.

### Task Design and Procedure

**Figure 1:**
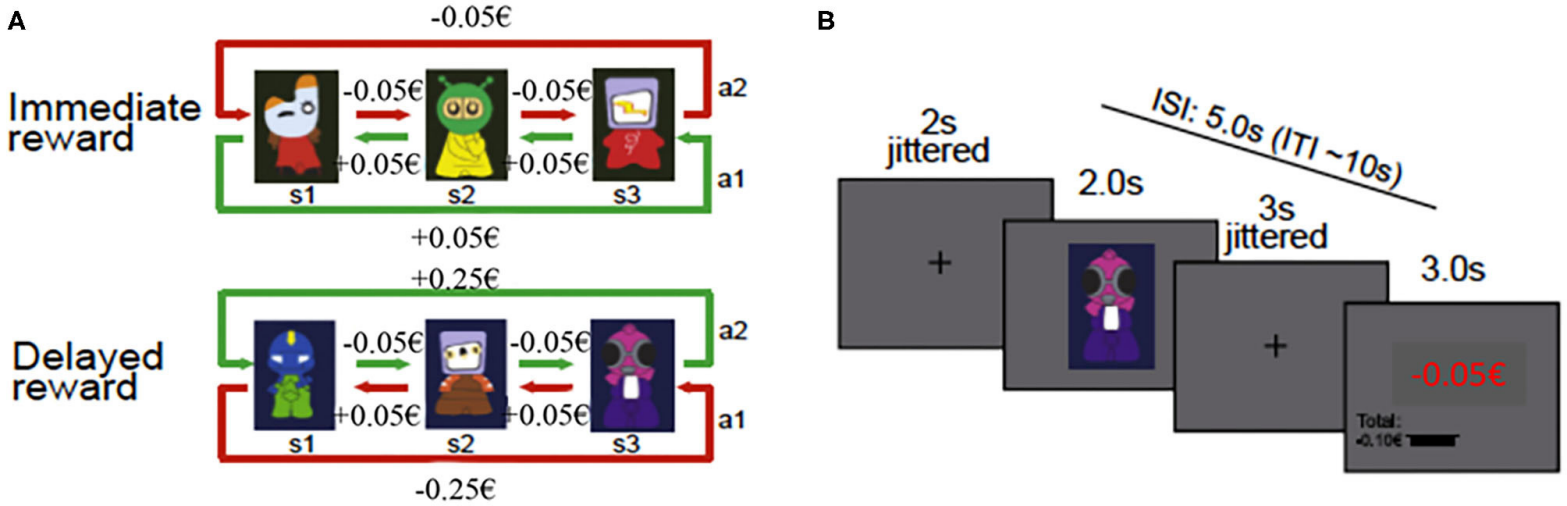
Schematic illustration of task design and trial procedure. **(A)** Task design of the three-state Markov decision task showing the two reward conditions (immediate and delayed) with the respective state transition structure (from Wittkuhn et al., 2018 Copyright 2020 by Elsevier). **(B)** Figure of the trial procedure (from Eppinger et al., 2015 Copyright 2020 by Elsevier). ISI, interstimulus interval; ITI, intertrial interval.

We used the three-state Markov decision task (see Figure 1A; Eppinger et al., 2015; Wittkuhn et al., 2018), which was programmed in EPrime 2.0 software (PST Inc., Pittsburgh, PA). In this task, an action at a specific state defines not only an outcome of this action but also determines the transition to the subsequent decision state (Tanaka et al., 2004; Eppinger et al., 2015). After presenting an abstract figure, participants had to press the left or right response button (Figure 1A). When the choice was made, participants received feedback concerning the financial amount received or lost in association with their decision. The task consisted of two reward conditions: (1) in the immediate reward condition, a consistent reward of five cents was paid for the optimal choice (as illustrated in green in Figure 1A) in all three states of the task, whereas a consistent loss of five cents resulted from all the other choices (as illustrated in red in Figure 1A); and (2) in the delayed reward condition, an optimal choice required the acceptance of small losses of five cents in the first two states which then led to a bigger reward of 25 cents in the third state (as illustrated in green in Figure 1A), whereas the suboptimal strategy resulted in small rewards of five cents in the first two states and a bigger loss of 25 cents in the third state (as illustrated in red in Figure 1A). Therefore, if optimal choices were made, a net win of 15 cents across the three states was expected irrespective of the reward condition.

The experiment consisted of a practice and an experimental phase. During the practice phase, a different combination of stimuli, decisions, and rewards was presented to familiarize participants with the task. Participants were instructed to ultimately maximize their reward and minimize their losses. No instruction concerning the existence of different conditions or possible strategies (e.g., to except small losses to receive a bigger reward) was given. The practice consisted of two blocks (one block per reward condition) and ended when the participants either reached 60% accuracy or completed a maximum number of 36 trials in the immediate reward condition or 72 trials in the delayed reward condition.

The experimental phase during fNIRS measurement consisted of the three-state Markov decision task that was comprised of eight blocks (four immediate reward blocks and four delayed reward blocks). In each block, consisting of 36 trials, a new set of three new stimuli was presented. Thus, the complete experimental task consisted of 288 trials in total (i.e., 144 trials in the immediate and 144 trials in the delayed reward condition). If a trial was missed, it was repeated until the response of the participant was received. The presentation of the reward conditions was conducted block-wise, the block order was randomized across participants, and the payoff rules were the same throughout all conditions. Each trial of the task started with the presentation of a fixation cross for a mean period of 2 s (jittered between 1 and 5 s). Then, the stimulus was presented for 2 s, followed by another fixation cross with a mean presentation time of 3 s, varying between 2 and 6 s (cf. Eppinger et al., 2015). Afterwards, the feedback for the current state, as well as the accumulated reward, was presented for 3 s (Figure 1B). The mean intertrial interval encompassed 10 s with a range of 8–16 s, resulting in approximately 50 min of the overall duration of the experimental task during fNIRS measurements.

### Study Design and Protocol of Transcranial Direct Current Stimulation

The tDCS experimental protocol was based on a double-blind between-subject design with tDCS and sham-stimulation conditions. tDCS and sham stimulation were performed offline based on the combined magnetic resonance spectroscopy and tDCS study of Bachtiar et al. (2015), showing that the most pronounced anodal tDCS induced inhibition of GABA neurotransmitter and concomitant increase in neural excitability occurs 15–30 min after the stimulation offset at rest.

**Figure 2:**
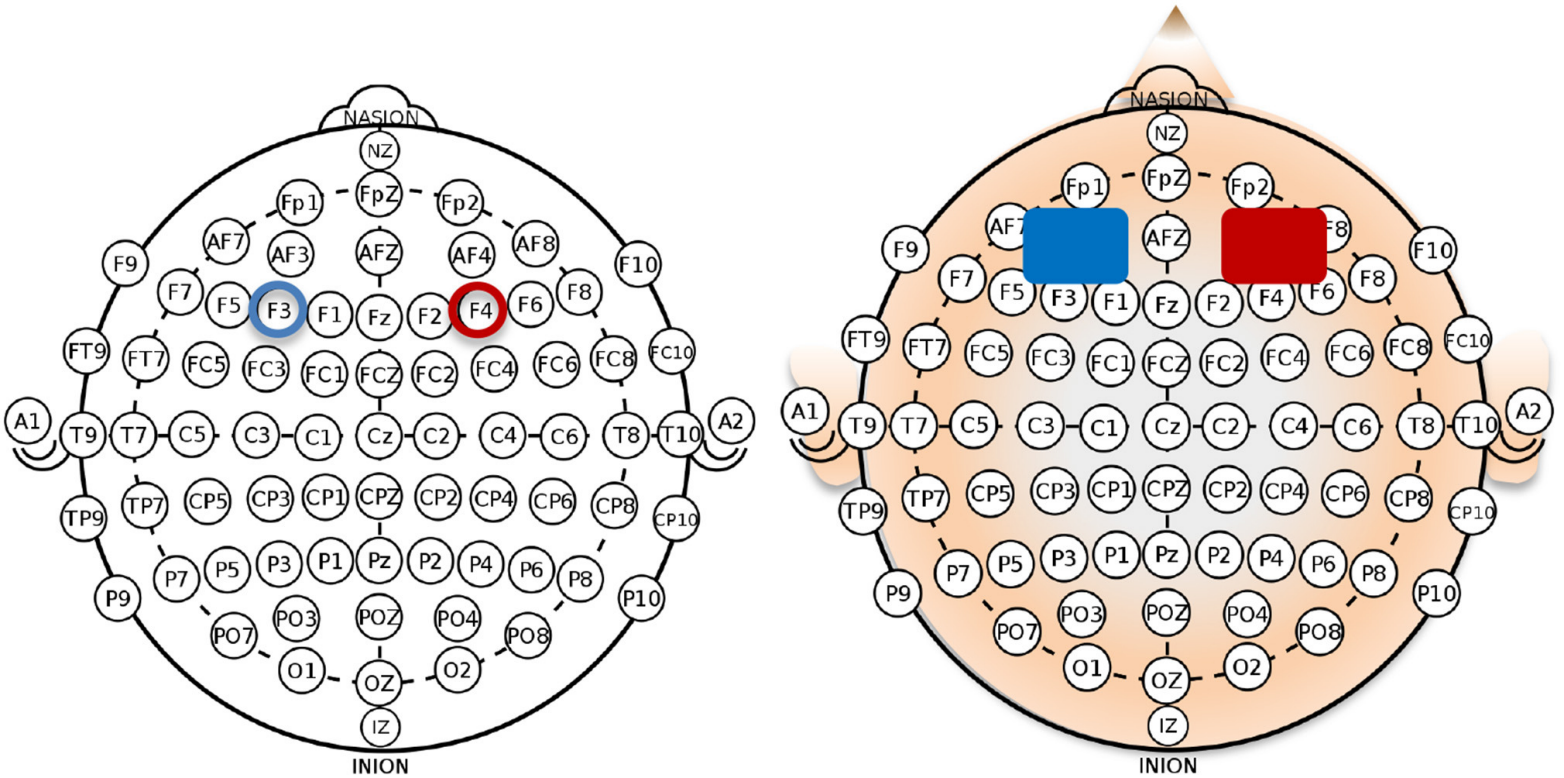
Right anodal/left cathodal montage for the application of bilateral transcranial direct current stimulation (tDCS) with the anode placed over the right dorsolateral prefrontal cortex (dlPFC; electrode position: F4 according to the 10–20 system) and the cathode over the left dlPFC (electrode position: F3).

The stimulation was conducted using a battery-driven current stimulator (neuroConn DC-Stimulator MR, neuroCare Group GmbH, Munich, Germany) and the current delivered through two sponge electrodes, soaked in saline to improve contact between the electrodes and the scalp (Figure 2). The total coverage area per electrode was 35 cm^2^ (5 cm × 7 cm each). The electrodes were placed bilaterally: anode over F4 corresponding to the right dlPFC and cathode over F3 corresponding to the left dlPFC, based on the international 10–20 EEG system (see Figure 2; Klem et al., 1999). tDCS consisted of 20-min right anodal stimulation with 15 s fade-in and 15 s fade-out for the total duration of the stimulation. The current intensity was set to a 2-mA constant current, which resulted in the current density of 0.057 mA/cm^2^. This stimulation protocol was chosen based on Fecteau et al. (2007b) as it produced an enhancing and stable effect over the bilateral dlPFC across participants. The sham stimulation was performed with the same electrode setup as the active stimulation; however, the 2-mA constant electrical current was delivered only for the first 30 s and then turned off, resulting in 0 mA stimulation for 19.5 min. The 30-s stimulation at the beginning was performed to induce the same sensations as during the actual tDCS stimulation (e.g., slight itching or tingling) which has been proven to be a successful method to keep participants blind concerning the stimulation condition (Brunoni et al., 2011).

### The Protocol of Functional Near-Infrared Spectroscopy

**Figure 3:**
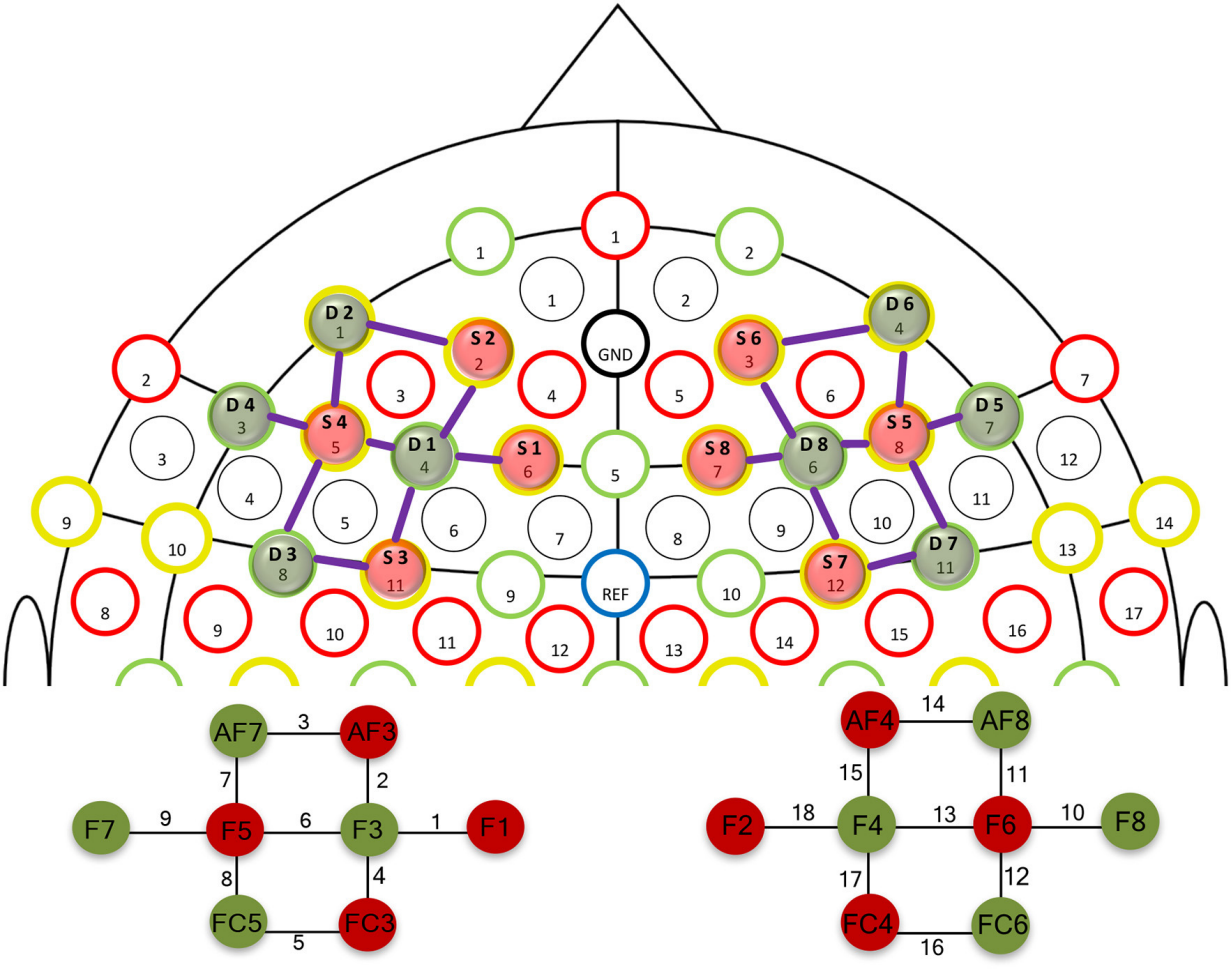
fNIRS optode setup for the left and right dlPFC according to the 10–20 system (Zimeo et al., 2018a).

The fNIRS data were recorded by using the continuous-wave battery-operated fNIRS system NIRSport (NIRx Medical Technologies, LLC, USA) that employs two distinct wavelengths (i.e., 760 and 850 nm) for the acquisition of intensity data. Eight illuminating sources and eight detection sensors arranged in a NIRScap according to the 10–10 EEG electrode placement were used to cover the left and right dlPFC (see Figure 3; Jurcak et al., 2007). This placement resulted in 18 measurement channels (as illustrated in Figure 3). The placement of the optodes was counterchecked based on AAL2 (Tzourio-Mazoyer et al., 2002; Rolls et al., 2015), Brodmann (Rorden and Brett, 2000), and Juelich (Eickhoff et al., 2005, 2006, 2007) anatomical landmark atlases with the help of fOLD Optodes' Location Decider (Zimeo et al., 2018a). The cross-coordinate check confirmed that the predefined cortical region of interest (ROI) corresponded to channels 1 (F1-F3), 2 (AF3-F1), 4 (FC3-F3), 6 (F5-F3), and 7 (F5-AF7) on the left side and channels 11 (F6-AF8), 13 (F6-F4), 15 (AF4-F4), 17 (FC4-F4), and 18 (F2-F4) on the right side (Figure 3).

The main limitation of this approach is the absence of subject-specific structural magnetic resonance imaging scans and a 3D digitizer (Selb et al., 2014; Tsuzuki and Dan, 2014), which precludes exact spatial co-registration of adapted subject's cortical coordinates to specific MNI coordinates. On the other hand, given the large extent of dlPFC activity captured by fNIRS (Ayaz et al., 2012a; Snowball et al., 2013; Fishburn et al., 2014; Choe et al., 2016; Vassena et al., 2019) and fNIRS spatial resolution, it is justifiable to assume that a finer anatomical localization based on fNIRS results would be difficult to attain. The source–detector separation distance was fixed to 3 cm in order to ensure an adequate depth sensitivity, taken the thickness of the human cranium of 5.4–8.2 mm (Li et al., 2007; Strangman et al., 2013). The data was sampled with a frequency of 7.81 Hz. After the tDCS application, the concentration changes in O_2_Hb and HHb were recorded during the performance of the three-state Markov decision task for approximately 50 min, including 2 min baseline measurement before and after the task execution to control for time-related drift rates in the data (Choe et al., 2016; Muthalib et al., 2016).

### Experimental Procedure

Participants filled in a sociodemographic questionnaire, the PANAS scale for pre-tDCS assessment, the BIS/BAS scale, and the Need for Cognition scale. Afterwards, they performed the Identical Pictures and Spot-a-Word Test as well as the WMT-2 Test. It took 45 min on average to complete the questionnaires and psychometric tests. Then, the bilateral RALC tDCS montage was set up and participants practiced the three-state Markov decision task for another 7 min. After the practice task, tDCS or sham stimulation was applied for 20 min. During the tDCS stimulation, the participant watched an excerpt from a silent nature documentary film *Unsere Wildnis* (Perrin and Cluzaud, 2016). When the tDCS stimulation was over, the three-state Markov decision task was conducted for approximately 50 min with simultaneous fNIRS recording.

### Data Analysis

#### Behavioral Data Analysis

Analysis of behavioral measures, i.e., response times (RTs) and optimal choice performance as well as psychometric (e.g., motivational traits, basic cognitive abilities, etc.) data, was computed with Matlab 9.3, R2017B (The MathWorks, Inc., MA, USA) and R packages (Version 3.4.3, R Core Team, 2012) in R Studio 1.1.414 (RStudio, Inc.). Mean RTs and the proportion of optimal choices per experimental condition (immediate and delayed reward) were calculated. Additionally, we separated between learning bins six consecutive equally sized trial bins reflecting learning within a block. The responses were averaged for each of these conditions (six bins for the immediate and the delayed reward condition each) per participant.

#### fNIRS Data Analysis

##### Data Preprocessing

The fNIRS data were preprocessed with the software analysis environment nirsLAB package (NIRx Medical Technologies, Glen Head, NY; Xu et al., 2014) based on Matlab (MATLAB, MathWorks Inc., Natick, MA, USA) and NIRS-SPM analysis packages (Ye et al., 2009). Concentration changes in O_2_Hb, HHb, and total hemoglobin (tHb = O_2_Hb + HHb) were analyzed and reported since it benefits a physiologically correct interpretation of the results in the absence of short-distance channel measurement in this study (Zimeo et al., 2018b). The coefficient of variation was calculated for each subject and each channel to control for signal quality. All channels with a coefficient of variation >15% were excluded from further analysis due to a high probability of the occurrence of non-physiological noise. The resting baseline intensity data was truncated (except for 15 s prior and 10 s after the experimental task) as not relevant for the analysis. No filtering was performed since, if applied, it might have falsely increased the anti-correlation between the time series (Murphy et al., 2009). Unfiltered intensity data was converted to optical density data and subsequently to O_2_Hb, HHb, and tHb changes applying the modified Beer–Lambert law (Cope and Delpy, 1988; Xu et al., 2014). The extinction coefficients set for 760 nm were 1.4866 (O_2_Hb) and 3.8437 (HHb), whereas for 850 nm, they were 2.5264 (O_2_Hb) and 1.7986 (HHb) in units of [(1/cm)/(mmol/L)]. To enhance the accuracy of the determined relative concentration changes, which is based on the age/wavelength interplay in the frontal head regions, differential pathlength factor (DPF) was calculated based on the mean age of 24 years for all subjects (Scholkmann and Wolf, 2013) resulting in 5.06 for O_2_Hb and 6.12 for HHb. For the baseline correction, the mean of the whole time series was chosen (Choe et al., 2016; Zimeo et al., 2018b) since it adds to the signal stability, attenuating the impact of irregular blood flow fluctuations (Tsunashima et al., 2012).

The general linear model (GLM) in the context of the canonical regression model has been solved with prewhitening AR(*n*) (not SPM based) analysis algorithm implemented in nirsLAB (Ye et al., 2009; Barker et al., 2013; Xu et al., 2014) to measure the channel-wise-evoked hemodynamic response and significant task-related cortical activation for each subject separately for O_2_Hb, HHb, and tHb and to correct for the main sources of confounding noise in fNIRS data. This prewhitening algorithm is based on filtering and “iteratively reweighted least squares” (Barker et al., 2013, p. 1378) and has shown excellent ability to correct for motion artifacts, attenuate the effects of serially correlated error, and estimate false-positive rates in fNIRS data (refer to Barker et al., 2013; Huppert, 2016 for more detailed information). Additionally, fNIRS data in each channel was addressed with different AR(*n*) (not SPM based) prewhitening filter. Regressors were generated by convolving the corresponding event time series with a canonical hemodynamic response function (HRF) for O_2_Hb and tHb and with an inverse of canonical HRF for HHb with a peak time set to 5 s, the time to reach its undershoot set to 16 s and with a maximum HRF duration of 32 s, accounting for a sluggish hemodynamic response (Benaron et al., 2000; Cazzell et al., 2012; Power et al., 2012; Mehnert et al., 2013). The peak time of 5 s for a canonical HRF was chosen based on prior fNIRS studies investigating hemodynamic correlates of cognitive functions in the dlPFC (Cazzell et al., 2012; Mehnert et al., 2013).

#### Statistical Analysis

The statistical analysis of fNIRS data was conducted using Matlab 9.3 R2017B and R packages in R Studio 1.1.414. The second-level analyses of the O_2_Hb, HHb, and tHb concentration changes were performed with the Statistical Parametric Mapping Level 2 of nirsLAB (Xu et al., 2014). For the within-subject factor *condition, t*-value maps were created for the contrast “delayed > immediate” based on the corresponding β values for each condition and measurement channel, using the two-sided *t*-test (*p* < 0.05, uncorrected for multiple comparisons) for each chromophore (O_2_Hb, tHb, and HHb), separately. For the between-group analysis (tDCS vs. sham stimulation), *t*-value maps were created for the contrast “tDCS > sham stimulation” based on the β values of both the immediate and the delayed reward condition, using the two-sided *t*-test (α ≤ 0.05, uncorrected for multiple comparisons) for each chromophore (O_2_Hb, tHb, and HHb), separately. Significant positive *t* values for O_2_Hb, tHb, and HHb indicated an increase in cortical activity reflecting an increase in O_2_Hb and a decrease in HHb, respectively.

We conducted linear mixed-effect models for performance measures (RTs and optimal choice performance) and the hemodynamic concentration changes (tHb, O_2_Hb, and HHb) using the lme function from the nlme package in R (Pinheiro et al., 2019). Linear mixed-effects models were calculated with maximum-likelihood estimation and *channel* as random intercepts nested into *subjects* [see Jasinska and Petitto (2013); Vassena et al. (2019) for a similar approach of crossed random effects to account for between-subject variability in hemodynamic concentration changes across individual channels] to analyze within-subject effects of the factor *condition* (i.e., immediate vs. delayed reward) and between-subject effects of the factor *treatment* (tDCS vs. sham stimulation). For the performance data, the model also considered within-subject effects of the factor *bin* (six bins per block) reflecting learning within a block. Results on these effects are presented in Results section of the Supplementary Material. For the data of the hemodynamic concentration changes, the models also considered within-subject effects of the factor *hemisphere*. Main effects of all models were followed up with *post-hoc* multiple comparisons using pairwise *t*-tests (Holm correction for multiple testing; Holm, 1979). According to the approach and recommendations by Fern and Monroe (1996) and Maxwell et al. (1981), we report partial eta-squared (η_p_^2^) for effect size estimations. When the models' residuals were not normally distributed as indicated by the Shapiro–Wilk test or the visual inspection using Q-Q plots, robust permutation tests were carried out. For this, we used a reduced model for the hemodynamic concentration changes data removing the factor *channel* and applied the lmer function from the lme4 package (Bates et al., 2015) and PERMANOVA from the predictmeans package in R (Luo et al., 2018). As the permutated models revealed similar results, we only report the effects initially estimated by the standard linear mixed-effects models. In order to determine the relationship between the modulation of the hemodynamic concentration changes by condition as an indicator of the upregulation of cortical activity in response to increasing task complexity on the one hand and performance in the more demanding experimental condition (delayed rewards) on the other hand, we carried out several correlational analyses. We calculated Pearson's product-moment correlation between the concentration modulation and RTs and Spearman's rank correlation coefficient for the concentration modulation and optimal choice performance (Holm correction for multiple testing, if not stated differently). The condition-dependent modulation of the hemodynamic concentration changes was defined as absolute change according to the formula: (*concentration*_*delayed*_−*concentration*_*immediate*_). For all analyses, a rejection criterion of *p* ≤ 0.05 was chosen.

## Results

### Bilateral tDCS Does Not Enhance Sequential Decision-Making Performance

**Figure 4:**
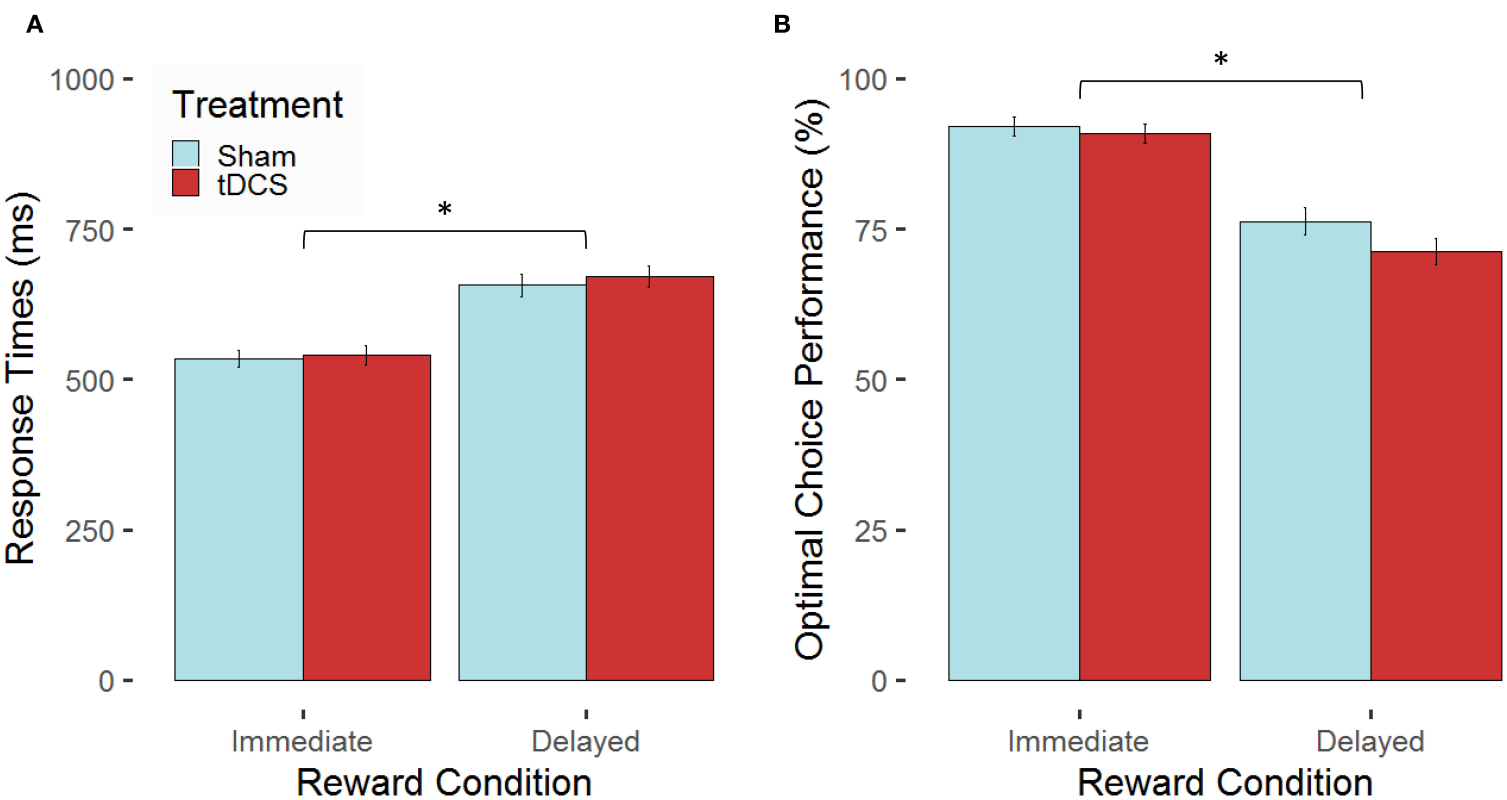
Stimulation-dependent modulation of behavioral performance for the two experimental conditions. Mean values (error bars indicate 1 standard error (SE) of the mean, *p < 0.05 for *post-hoc* multiple comparisons after Holm correction) are indicated for **(A)** response times in milliseconds (ms) and **(B)** the proportion of optimal choices in percent (%) both separated for the two experimental conditions (immediate vs. delayed reward) for participants, who received bilateral active transcranial direct current stimulation (tDCS; red) compared with sham stimulation (blue).

First, findings on potential tDCS-related changes in decision time and optimal choice behavior are reported. We expected performance-enhancing tDCS effects in terms of faster and more optimal decisions after tDCS compared with sham stimulation. The linear mixed-effects model for RTs revealed a main effect of *condition, F*_(1, 297)_ = 166.09, *p* < 0.0001, η_p_^2^ = 0.36, whereas the main effect of *treatment, F*_(1, 27)_ = 0.07, *p* = 0.79, and the interaction *condition* × *treatment, F*_(1, 297)_ = 0.18, *p* = 0.67, did not reach significance. Participants decided faster in the immediate reward condition compared with the delayed reward condition (see Figure 4A). The linear mixed-effects model for the proportion of optimal choices revealed likewise a main effect of *condition, F*_(1, 297)_ = 167.68, *p* < 0.0001, η_p_^2^ = 0.36. Again, the main effect of *treatment, F*_(1, 27)_ = 0.85, *p* = 0.37, and the interaction *condition* × *treatment, F*_(1, 297)_ = 1.19, *p* = 0.17, did not reach significance. Participants made more optimal choices in case of immediate compared with delayed reward (see Figure 4B). A complete overview comprising the statistics of all effects of the applied models on performance data can be found in the Supplementary Material (see Supplementary Results; Supplementary Table 1).

### tDCS Effects on the Hemodynamic Response

In the following, the hemodynamic correlates of sequential decision-making and potential tDCS-induced effects at the brain level are presented. We expected a condition-specific modulation of the neural response in terms of elevated tHb, O_2_Hb, and HHb concentration levels for the delayed compared with the immediate reward condition. Further, we assumed a higher neural response after tDCS compared with sham stimulation. The linear mixed-effects model for the tHb concentration changes revealed the main effects of *treatment, F*_(1, 27)_ = 4.35, *p* < 0.05, and *condition, F*_(1, 518)_ = 6.26, *p* = 0.01, η_p_^2^ = 0.01, as well as a significant interaction *treatment* × *hemisphere, F*_(1, 491)_ = 9.46, *p* = 0.002, η_p_^2^ = 0.02. The interaction effect of *condition* × *treatment* × hemisphere just failed to reach significance, *F*_(1, 518)_ = 3.16, *p* = 0.08. There was no significant main effect of *hemisphere, F*_(1, 491)_ = 0.28, *p* = 0.60. The interaction effects *condition* × *treatment, F*_(1, 518)_ = 0.01, *p* = 0.91, and *condition* × *hemisphere, F*_(1, 518)_ = 0.80, *p* = 0.37, also did not reach significance. Similar to these findings, the linear mixed-effects model for the O_2_Hb concentration revealed a main effect of *condition, F*_(1, 518)_ = 10.52, *p* = 0.001, η_p_^2^ = 0.02, and a significant interaction of *treatment* × *hemisphere, F*_(1, 491)_ = 7.01, *p* = 0.008. The main effect of *treatment, F*_(1, 27)_ = 3.71, *p* = 0.06, and the interaction of *condition* × *treatment* × *hemisphere, F*_(1, 518)_ = 3.07, *p* = 0.08, both just failed to reach significance. There was no significant main effect of *hemisphere, F*_(1, 491)_ = 0.19, *p* = 0.66. The interaction effects of *condition* × *treatment, F*_(1, 518)_ = 2.07, *p* = 0.15, and *condition* × *hemisphere, F*_(1, 518)_ = 2.36, *p* = 0.13, did not reach significance as well. The linear mixed effects model for the HHb concentration changes revealed a main effect of *condition, F*_(1, 518)_ = 5.77, *p* = 0.02, η_p_^2^ = 0.01. The main effect of *hemisphere* just failed to reach significance, *F*_(1, 491)_ = 1.97, *p* = 0.08. The main effect of *treatment, F*_(1, 27)_ = 1.01, *p* = 0.32, did not reach significance either. There were also no significant interaction effects, neither for *condition* × *treatment, F*_(1, 518)_ = 0.23, *p* = 0.63, nor for *condition* × *hemisphere, F*_(1, 518)_ = 0.06, *p* = 0.81, *treatment* × *hemisphere, F*_(1, 491)_ = 0.26, *p* = 0.61, or *condition* × *treatment* × *hemisphere, F*_(1, 518)_ = 0.13, *p* = 0.72.

**Figure 5:**
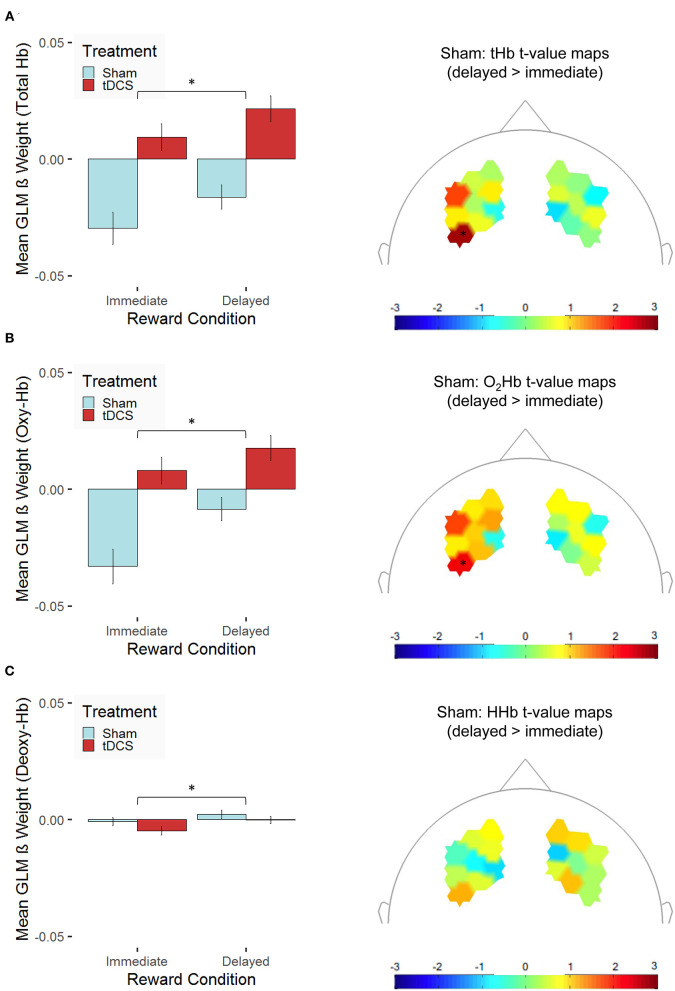
Hemodynamic concentration changes in the dorsolateral prefrontal cortex are presented for **(A)** total hemoglobin (tHb), **(B)** oxygenated hemoglobin (O_2_Hb/Oxy-Hb), and **(C)** deoxygenated hemoglobin (HHb/Deoxy-Hb). The left panel presents mean β values [error bars indicate 1 standard error (SE) of the mean, *p < 0.05 for *post-hoc* multiple comparisons after Holm correction] across all channels separated for the two experimental conditions (immediate vs. delayed reward) for participants, who received bilateral active transcranial direct current stimulation (tDCS; red) compared with sham stimulation (sham; blue). To prove whether functional near-infrared spectroscopy (fNIRS) is suitable to capture condition-specific activation patterns as functional magnetic resonance imaging (fMRI) does, the right panel indicates *t*-value maps depicting the contrast between the experimental conditions (delayed > immediate reward) only for participants, who received sham stimulation, unthresholded (* indicates thresholded *t* values with *p* < 0.05 before correcting for multiple testing; none of these values survives Holm correction).

Following up these main and interaction effects of the three models using pairwise *t*-tests revealed that, as expected, the tHb concentration changes in the dlPFC were higher for delayed rewards compared with immediate rewards (see Figure 5A). This effect was present for participants of both groups: in the sham-stimulation group, which replicates findings of the fMRI study by Eppinger et al. (2015) and also in the tDCS group (see condition-specific β weights separated for groups in Figure 5A, left). Similarly, there were higher O_2_Hb concentration changes (see Figure 5B) and higher HHb concentration changes (see Figure 5C) in the delayed reward condition compared with the immediate reward condition. The spatial distributions of the condition-related differences are illustrated in the topographic maps of Figure 5. However, one has to note that none of the channel-related *t* values reaches significance after correcting for multiple testing (Holm correction). Our findings only allow for clear statements on a general condition effect on the hemodynamic response.

**Figure 6:**
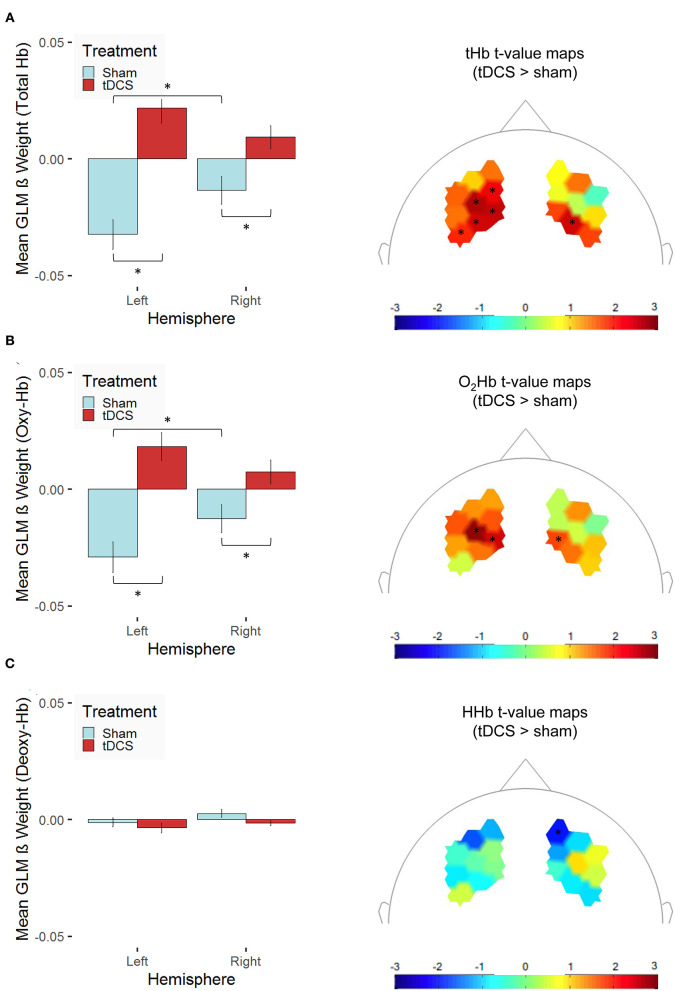
Stimulation-dependent modulation of hemodynamic concentration changes in the dorsolateral prefrontal cortex is shown for **(A)** total hemoglobin (tHb), **(B)** oxygenated hemoglobin (O_2_Hb/Oxy-Hb), and **(C)** deoxygenated hemoglobin (HHb/Deoxy-Hb). The left panel indicates mean β values [error bars indicate 1 standard error (SE) of the mean, *p < 0.05 for *post-hoc* multiple comparisons after Holm correction] across the channels of the two hemispheres (left vs. right) averaged across both experimental conditions for participants, who received bilateral active transcranial direct current stimulation (tDCS; red) compared with sham stimulation (sham; blue). The right panel presents *t*-value maps depicting the contrast between the two treatment groups (tDCS > sham stimulation), unthresholded (* indicates thresholded *t* values with *p* < 0.05 before correcting for multiple testing; none of these values survives Holm correction).

Further, tHb concentration changes were elevated in the right compared with the left hemisphere, but only in the sham-stimulation group, *t*_(251)_ = 2.61, *p* = 0.01, and not in the tDCS group, *t*_(269)_ = −1.81, *p* = 0.07, where—at the trend level—the opposite pattern occurred. As we hypothesized, tHb concentration changes were higher after bilateral tDCS compared with sham stimulation in both hemispheres [left: *t*_(519.09)_ = 6.10, *p* < 0.001; right: *t*_(500.05)_ = 2.93, *p* = 0.004; see Figure 6A]. This finding might be indicative of tDCS-induced modulation of the neural response during sequential decision-making. Similarly, O_2_Hb concentration changes were elevated in the right compared with the left hemisphere, but only in the sham-stimulation group, *t*_(251)_ = 2.35, *p* = 0.02, and not in the tDCS group, *t*_(269)_ = −1.58, *p* = 0.12. At the same time, we could not observe any treatment-related modulation of the HHb concentration changes. In both hemispheres, O_2_Hb concentration changes [left: *t*_(511.77)_ = 5.32, *p* < 0.001; right: *t*_(501.82)_ = 2.54, *p* = 0.01; see Figure 6B] but not HHb concentration changes (see Figure 6C) were higher after bilateral tDCS compared with sham stimulation. The spatial distributions of the treatment-related differences are illustrated in the topographic maps of Figure 6. However, one has to note that none of the channel-related *t* values reaches significance after correcting for multiple testing (Holm correction). Our findings allow for clear statements on a treatment effect on the hemodynamic response differentiated for the two hemispheres only.

### Relationship Between Performance and Hemodynamic Response

**Figure 7:**
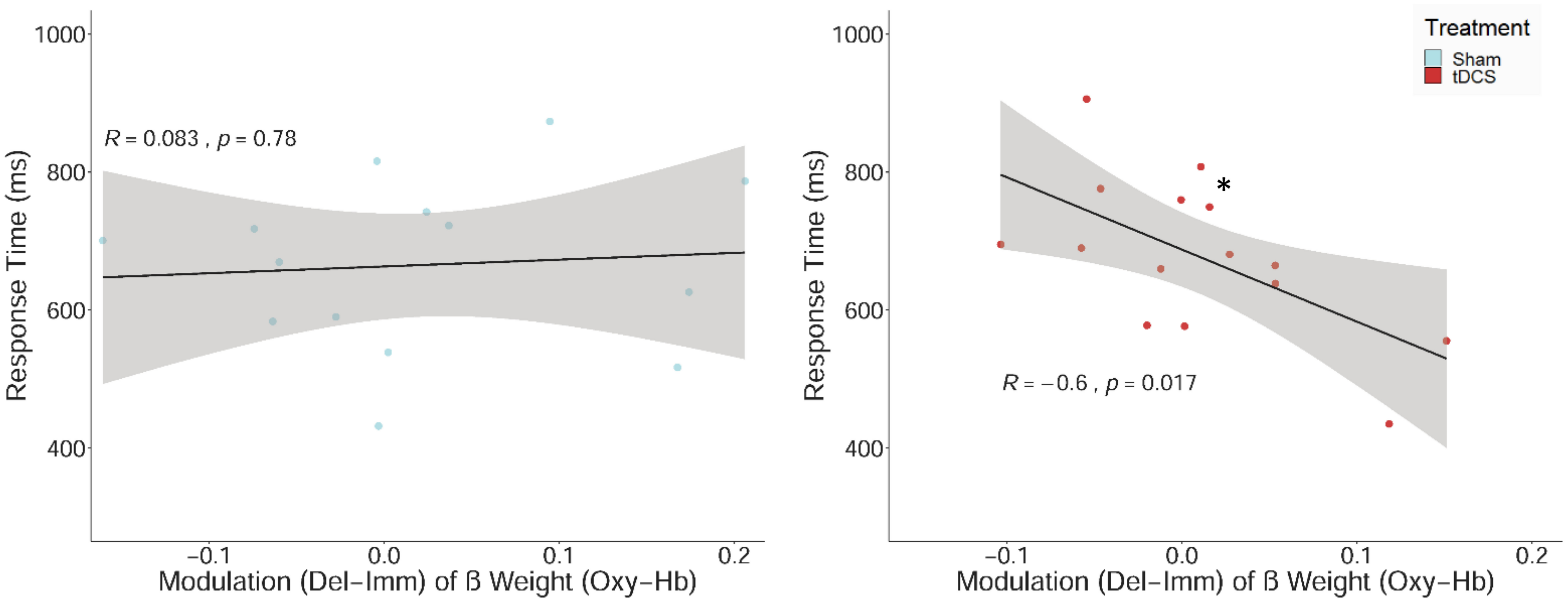
Scatterplot showing the relations between the upregulation of the hemodynamic response (β values) between the two experimental conditions (delayed–immediate reward; Del-Imm) and response times in milliseconds (ms) in the delayed reward condition. Correlation including regression lines with Pearson's correlation coefficient (*R*), *p*-value (*p < 0.05), and confidence region (95%; gray area are separately shown for participants, who received sham stimulation (sham; left panel) and bilateral active transcranial direct current stimulation (tDCS; right panel).

Lastly, we expected neuromodulatory effects, i.e., the condition-specific upregulation of the neural response during decision-making would be positively associated with performance in the delayed reward condition. The correlation analyses of the condition-dependent modulation of the hemodynamic response and performance measures in the delayed reward condition revealed a significant negative relationship between the condition-dependent upregulation of O_2_Hb and RTs (see Figure 7). This association was present after receiving tDCS (*r* = −0.60, *p* = 0.03; not corrected for multiple comparisons) but not after sham stimulation (*p* = 0.78). Although the negative correlation in the tDCS group did not survive Holm correction for multiple testing, the difference between the correlations of the two treatment groups (tDCS vs. sham stimulation) turned out to be significant (*z* = 1.86, *p* = 0.03; Fisher *r*-to-*z* transformation).

All the other correlations did not reach significance (see Table 2).

**Table 2:** Results of neural–behavioral correlation.

**Hemodynamic response**	**tDCS**		**Sham stimulation**	
	**Correlation**	***p*-value**	**Correlation**	***p*-value**
tHb—RTs	−0.48	0.07 (0.77)	0.11	0.71 (1.0)
tHb—opt. choices	0.23	0.42 (1.0)	−0.48	0.09 (0.90)
O_2_Hb—RTs	−0.60	0.02 (0.24)	0.08	0.78 (1.0)
O_2_Hb—opt. choices	0.33	0.23 (1.0)	−0.34	0.24 (1.0)
HHb—RTs	−0.18	0.52 (1.0)	0.16	0.59 (1.0)
HHb—opt. choices	0.26	0.35 (1.0)	−0.40	0.15 (1.0)

*Statistical values are shown for the correlation analyses between the condition-dependent modulation of hemodynamic concentration changes for total hemoglobin (tHb), oxygenated HB (O_2_Hb) as well as deoxygenated HB (HHb), and response times (RTs; correlation coefficient: Pearson's r) as well as optimal choice performance in the delayed reward condition (opt. choices; correlation coefficient: Spearman's ρ) separated for participants, who received active bilateral transcranial direct current stimulation (tDCS) compared with sham stimulation (sham)*.

*p-value uncorrected and after Holm correction for multiple testing in brackets*.

## Discussion

In the present study, we investigated the neural correlates of stimulation-induced modulation of the ability to learn the value of future outcomes and the sequential choices necessary to achieve them (i.e., sequential decision-making). For this purpose, we combined bilateral tDCS with fNIRS and used a three-state Markov decision task that consisted of two (i.e., immediate and delayed) reward conditions. The results showed (i) enhanced dlPFC hemodynamic response during the acquisition of sequential state transitions in line with the findings of a previous fMRI study showing similar neural activation patterns; (ii) tDCS-induced increase of hemodynamic response in the dlPFC irrespective of reward condition, but without performance enhancement at the behavioral level; and (iii) tDCS-related modulation of the hemodynamic response seems to be related to faster response times in the delayed reward condition. Taken together, the findings provide empirical evidence that fNIRS is a suitable method to investigate the neural correlates of sequential decision-making and shed further light on the underlying neural correlates of tDCS-induced effects.

### fNIRS Is a Suitable Method for Measuring Neural Correlates of Sequential Decision-Making

To investigate the neural correlates of sequential decision-making with and without tDCS-induced effects, we employed fNIRS during a three-state Markov decision task performed by a corresponding tDCS and sham-stimulation group. In the latter group, we were able to explore our proof-of-concept idea that fNIRS is capable to capture the differences in hemodynamic response between the immediate and delayed reward conditions in a three-state Markov decision task similar to an earlier fMRI study, both having comparable participant groups (Eppinger et al., 2015). Consistent with our expectations, the results based on the data from the sham-stimulation group revealed significantly higher O_2_Hb and tHb with a corresponding significant decrease in HHb concentration changes, indicating greater recruitment of the lateral PFC in the delayed reward condition in comparison with the immediate reward condition. Critically, the results of the present study are consistent with the abovementioned fMRI study that used the same task and showed a greater blood oxygenation level-dependent response (i.e., enhanced recruitment) of the lateral PFC regions in the delayed compared with the immediate reward condition in young adults (Tanaka et al., 2004; Eppinger et al., 2015). Such enhanced recruitment of the lateral PFC may reflect the learning of the state transition structure of the task to predict future (i.e., delayed) rewards in order to reach an optimal overall result (Tanaka et al., 2004; Durstewitz et al., 2010; Lee et al., 2014; Eppinger et al., 2015; Friedrich and Lengyel, 2016; Wittkuhn et al., 2018). In the field of sequential decision-making, we demonstrate that the application of fNIRS provides evidence consistent with fMRI studies, which extends earlier findings for risk decision-making, task preparation, and execution (Ayaz et al., 2012b; Cazzell et al., 2012; Mehnert et al., 2013; Vassena et al., 2014, 2019; Causse et al., 2017; Di Rosa et al., 2019; McKendrick et al., 2020). Thus, the present study also holds novel methodological implications. In addition, similar condition-specific differences in O_2_Hb and tHb concentration changes in the dlPFC were also shown in the tDCS group of the present study, indicating that the direction of neural response patterns for the three-state Markov decision task abides with tDCS-induced neurostimulation. Taken together, the current proof-of-concept results provide empirical evidence that fNIRS is a suitable cost-effective and non-invasive method to measure condition-dependent hemodynamic correlates (i.e., O_2_Hb, HHb, and tHb concentration changes) of sequential decision-making during a three-state Markov decision task irrespective of tDCS application.

This finding is especially important in the light of the growing interest for repetitive cognitive trainings (with or without tDCS) aimed to counteract deficits in and enhance different cognitive functions (Park et al., 2014; Katz et al., 2017; Martin et al., 2017; Lawrence et al., 2018; Nissim et al., 2019), in particular sequential decision-making (Antonenko et al., 2019; Thams et al., 2020b) in patients and older populations. fNIRS may allow cost- and time-effective monitoring of hemodynamic correlates of such interventions, aiming to improve their efficiency through repeated investigation and analysis of individual neural responsiveness. fNIRS has been already successfully used for this purpose in research in natural environments [e.g., assessing dual tasks performance while walking and executing an arithmetic task (Lu et al., 2015), cognitive control while playing a simple game task (Witte et al., 2015), skill acquisition during repeated flight simulator training and n-back working memory tasks (Choe et al., 2016), and decision-making and sustained attention in marine evasive maneuver task (Fan et al., 2020) among others]. Therefore, fNIRS may be considered as a suitable alternative to other neuroimaging techniques (e.g., fMRI, computed tomography) for cognitive intervention research and in future studies addressing sequential decision-making.

In order to improve the monitoring quality of fNIRS measurements in future studies, auxiliary physiological signals should be accounted for. In order to control for systemic effects and non-neuronal-driven hemodynamic changes (e.g., blood pressure, heart rate, breathing rate, changes due to carbon dioxide concentration, etc.) in fNIRS measurements (Tak and Ye, 2014; Tachtsidis and Scholkmann, 2016), future studies should incorporate synchronous monitoring of systemic physiology with appropriate instrumentation and include this data into the analysis. Additionally, short-distance channel measurements that are more sensitive to extracerebral hemodynamic concentration changes (Tak and Ye, 2014; Zhang et al., 2015; Zimeo et al., 2018b) should be incorporated to identify and exclude task-related systemic changes that may confound measured functional responses. Furthermore, subject-specific magnetic resonance imaging scans and a 3D digitizer should be included to ensure exact spatial co-registration (Selb et al., 2014; Tsuzuki and Dan, 2014). This together with anatomical landmark atlases used in this study (Zimeo et al., 2018a) will help to ensure more precise localization of hemodynamic changes. Future studies may consider an additional pre-tDCS baseline fNIRS measurement as well as fNIRS measurement during tDCS application in addition to post-tDCS hemodynamic monitoring in tDCS and control groups (Di Rosa et al., 2019). Last but not least, the placement of additional fNIRS measurement optodes to get a better understanding of tDCS-induced modulation and its neural extend should be also considered (Zimeo et al., 2018b). The next section presents the results on behavioral and neural tDCS-induced modulation of sequential decision-making.

### Bilateral tDCS Increases Hemodynamic Response in Both Reward Conditions but Does Not Enhance Choice Performance

To investigate behavioral and neural modulation of sequential decision-making, we employed bilateral offline tDCS over the dlPFC before the execution of the three-state Markov decision task while simultaneously recording fNIRS. According to our expectations, compared with sham stimulation, tDCS elevated hemodynamic response in the lateral PFC during sequential decision-making. This excitatory effect is in line with tDCS-induced increases in O_2_Hb concentration changes in the dlPFC during a working memory task (Jones et al., 2015), in the right dlPFC during a Balloon Analog Risk Task (Weber et al., 2014), in the left PFC during a reward-based working memory task (Di Rosa et al., 2019), and at rest (Merzagora et al., 2010; Muthalib et al., 2018), with the high-definition tDCS-induced change of O_2_Hb concentration in the left motor cortex (Muthalib et al., 2018) and with high-definition tDCS-induced changes in O_2_Hb and HHb in the dlPFC and left ventral medial PFC during a spatial working memory task (McKendrick et al., 2020). Therefore, the elevated hemodynamic response in the lateral PFC observed in our study points out at the neural excitatory impact of the offline tDCS during sequential decision-making, supporting and extending previous research findings. However, the interpretation of our findings is limited to the healthy younger male adult population.

In the present study, a tDCS-induced excitatory effect was observed across both reward conditions. Similarly, Jones et al. (2015) showed tDCS-induced increases in O_2_Hb concentration changes in both experimental conditions (low vs. high motivation) during a working memory task, and Di Rosa et al. (2019) showed bilateral activation not only in the tDCS offline period but also during tDCS. The condition-independent increase in O_2_Hb and tHb and decrease in HHb may be attributed to the direct impact of tDCS on the dilation of blood vessels due to changes in astrocyte activity which produces global hemodynamic changes as suggested by previous findings (Bikson et al., 2004; Ruohonen and Karhu, 2012; Takai et al., 2016). Furthermore, the tDCS-induced excitatory effect in the dlPFC was observed in both hemispheres. As it was shown, prefrontal tDCS may also affect both activity and resting-state connectivity of regions inside and outside stimulated sites that are relevant for the task (Weber et al., 2014; Takai et al., 2016; Möller et al., 2017). For example, Weber et al. (2014) could show that bilateral tDCS over the dlPFC during a Balloon Analog Risk-Taking Task influenced both fronto-frontal and fronto-striatal functional connectivity, modulating resting and task-related activity in the PFC, dorsal striatum, and posterior regions of the cortex. Anodal tDCS may also enhance hemodynamic response not only in the stimulated areas recruited by the task but also in those not directly involved but highly interconnected brain regions (Di Rosa et al., 2019). Overall, these findings are in line with the research on tDCS neural effects, showing excitatory neural effect (Bachtiar et al., 2015; Jones et al., 2015; Antonenko et al., 2017; Di Rosa et al., 2019).

One has to bear in mind that a bilateral electrode setup as used in the present study leads to a bipolar stimulation. With this type of stimulation, we cannot distinguish between anodal and cathodal effects nor differentiate which region has been affected the most (Nitsche et al., 2007, 2008). Nevertheless, the methodological combination of tDCS and fNIRS could provide first information about the localization of stimulation effects albeit the more restricted spatial resolution of fNIRS in comparison with fMRI. The excitatory effect in both hemispheres together with the reverse relation between right and left neural activation after stimulation in the present study suggests potential stimulation-induced changes in the hemispheric interplay. However, the current data do not allow us to differentiate which exact regions have been affected. The NIRS montage covered prefrontal regions including the dlPFC, but channel-specific effects could not have been analyzed. Similarly, the detection of potential small modulatory effects for single channels requires greater power. Accordingly, none of the channel-specific *t* values on concentration-level changes reached significance after correcting for multiple comparisons (see Figure 6). Besides recruiting larger samples for cost-effective studies combining tDCS with fNIRS, future studies that investigate functional brain mechanisms underlying tDCS-induced enhancement of sequential decision-making should also lean on MRI to substantiate and extend the findings of the present study.

Individual anatomical peculiarities such as the topography of the cortical surface, orientation of the cortical pyramidal neurons, level of fat, cerebrospinal fluid density, and skull thickness may significantly and differently impact the flow and density of the tDCS-induced electrical currents in a variety of ways despite the same stimulation protocol, resulting in different tDCS-induced impact (Rahman et al., 2013; Truong et al., 2013; Kim et al., 2014; Dutta et al., 2015; Woods et al., 2019; Filmer et al., 2020; Habich et al., 2020). Sex-specific morphological differences should also be taken into consideration since they were shown to differentiate performance-related tDCS effects (León et al., 2020). The same applies to age-specific neurochemical, functional, and morphological differences (Antonenko et al., 2017, 2019). Moreover, intra- and interindividual differences in neurotransmitter concentration (e.g., in dopamine, GABA, glutamate) as well as concentration and genotype of brain-derived neurotrophic factor or catechol-O-methyl transferase may lead to distinct tDCS-induced modulatory neural and behavioral effects (Teo et al., 2014; Li et al., 2015; Nitsche et al., 2015; Stephens et al., 2017; Horne et al., 2020). For instance, Horne et al. (2020) reported a transfer effect in visual episodic memory in Val/Val carriers of aforementioned genes after tDCS coupled with decision-making training, whereas no beneficial training or transfer effect was observed in carriers of other genotypes. The aforementioned aspects may lead to distinct responsiveness to tDCS across individuals resulting in a set of responders and non-responders and an overall null effect (López-Alonso et al., 2014; Wiethoff et al., 2014; Nejadgholi et al., 2015). Therefore, individualized and adjusted stimulation protocols (Berker et al., 2013; Edwards et al., 2013; Li et al., 2015; Nejadgholi et al., 2015; Laakso et al., 2016; Stephens et al., 2017) should be considered for future research to ensure the efficacy of tDCS interventions. Moreover, more precise electrode montages (e.g., high-definition tDCS, multi-array electrodes) should be applied (Laakso et al., 2016), as it was shown that current density and focality of the tDCS effect improved with the smaller size of the electrodes (Faria et al., 2011). Recent research supports these claims, illustrating how the different numbers of tDCS interventions and distinct tDCS protocols and montages can be associated with different outcomes (Horne et al., 2020). For example, Hanley and Tales (2019) showed an improved attentional control in older adults after three sessions of left anodal and right cathodal tDCS (1.5 mA, 20 min stimulation duration) over the dlPFC, whereas Nilsson et al. (2017) demonstrated evidence against a beneficial effect of 20 sessions of left anodal tDCS over dlPFC (2 mA, 25 min stimulation duration) on working memory performance. Therefore, future studies should also focus on individually fine-tuned stimulation protocols and stimulation dosage (frequency, strength, etc.) to provide efficient tDCS interventions that are safe in the long run.

Concerning the performance-related effect of tDCS, contrary to our expectations, bilateral tDCS over the dlPFC did not have a performance-enhancing effect during sequential decision-making. Participants who received tDCS did not show enhanced learning of the state transitions compared with participants who received sham stimulation. These results are in contrast to previous findings indicating enhancing effects of bilateral tDCS over the dlPFC for various kinds of decision-making processes (Fecteau et al., 2007a,b; Minati et al., 2012; Jones et al., 2015; Choe et al., 2016; Edgcumbe et al., 2019; Soyata et al., 2019; McKendrick et al., 2020). However, they are in line with the observed tDCS-induced excitation of neural activity patterns without significant performance-related effects of tDCS shown by Möll (2015) and Weber et al. (2014). Herrmann et al. (2017) also reported a missing link between tDCS-induced neural and behavioral modulations during a verbal fluency task. Performance facilitation but not performance enhancement was also shown by Di Rosa et al. (2019), after left anodal tDCS during the reward-based working memory task. An initial high level of performance observed in younger adults may also be a potential explanation for the tDCS null effect in the present study. Eppinger et al. (2015) reported a rapid decline of dlPFC activity as soon as participants get the insight into the state transitions. At this stage, bilateral tDCS over a dlPFC might not have the potential to further alter task performance. This consideration is also in accordance with the so-called notion of neural efficiency (Neubauer and Fink, 2009). Novel cognitive tasks with sufficient practice time lead to the development of efficient strategies, thereby decreasing the demand for neural resources. For instance, Ayaz et al. (2012a) showed that the focused practice on an ecological cognitive task reduced neural response of the left dlPFC. In line with this claim, Di Rosa et al. (2019) reported no enhancing effect during working memory task when the baseline performance of the participant was high, referring to already existing optimal performance level prior to tDCS. The sample size or the composition of our sample may also account for our results. Interestingly, even in a large-scale study including 200 participants, Russo et al. (2017) reported a null effect of bilateral tDCS on risk decision-making in the Balloon Analog Risk Task. The authors argue that tDCS effects observed in several studies investigating the stimulation-induced modulation of different cognitive functions might stem from high interindividual variability in response to tDCS. An observed null effect in our study may also be attributed to high interindividual differences in response to tDCS, which may be seen in the within-group task-related performance variability. Despite this interindividual variability in response to tDCS, Hsu et al. (2016) showed also a high intraindividual consistency in these response patterns, indicating that a cluster analysis approach could provide deeper insights into the null effect of tDCS. In addition, Filmer et al. (2020) showed that averaging across groups leads to the loss of individual information about tDCS effects that depend on individual baseline neurochemicals and cortical morphology. The clustering approach based on the information combination on individual neurochemicals, neural architecture, function, and behavior may help to shed light on the interaction of these factors with respect to tDCS effect and increase its efficacy and improve the adjustment of tDCS protocols (Filmer et al., 2020). The sample size of the present study (*n* = 29) precluded this approach (Formann, 1984; Dolnicar et al., 2014). Future studies with larger sample sizes might be able to identify subsamples of non-responders and positive and negative responders and draw more robust conclusions of potential enhancing tDCS-induced effects on sequential decision-making performance.

Despite an extensive body of research dedicated to the understanding of tDCS neuromodulatory effects and high public interest in the face of current demographic development, still little is known about the exact interplay of factors that lead to the described excitatory effects (Utz et al., 2010; Richardson et al., 2019; Habich et al., 2020), making the generalization, transfer, and interpretation of tDCS cumbersome (Woods et al., 2019). This applies also for our study despite adopting a probed RALC tDCS protocol which was shown to augment cognitive performance (Fecteau et al., 2007a; Edgcumbe et al., 2019; Soyata et al., 2019). Notwithstanding the null effect regarding tDCS-induced enhancement of sequential decision-making in the present sample of young male adults, tDCS could be a potential tool to enhance decision-making performance in older adults. Eppinger et al. (2015) show an under-recruitment of the lateral PFC in older adults that was associated with deficits in learning to predict future rewards. Therefore, the tDCS-induced elevation of the hemodynamic response in lateral PFC observed in the current study may be a potential neuromodulatory tool to counteract the deficits of decision-making performance in older adults. The modulation of decision speed as one such aspect will be discussed in the following section.

### tDCS-Induced Neural Upregulation Is Related to Facilitated Decision Speed in the Delayed Reward Condition

In the present study, condition-dependent (i.e., delayed–immediate) upregulation of O_2_Hb concentration changes seems to be negatively associated with RT in the tDCS but not in the sham-stimulation group. The correlation coefficients of both groups were significantly different from each other. This negative neural–behavioral correlation only in the tDCS group suggests that participants seem to differ in their response to tDCS as discussed above. More specifically, participants with a higher upregulation effect also seem to perform faster in the delayed reward condition of this task (i.e., positive tDCS responders), whereas a lower upregulation seems to be associated with slower task performance (i.e., negative or tDCS non-responders), similar to findings reported by earlier studies (Nejadgholi et al., 2015; Russo et al., 2017; Di Rosa et al., 2019; Lefebvre et al., 2019). The neural–behavioral associations based on optimal choice performance did not reach significance, indicating that the observed tDCS-induced hemodynamic upregulation was not accompanied by more successful learning to predict future reward. These findings are in line with previous evidence, showing that a single tDCS application often is accompanied by performance facilitation only expressed through faster response times (Horvath et al., 2014; Dedoncker et al., 2016). Although we applied only one session of offline tDCS with healthy younger adult male participants, it is important to take into consideration a wider scope of similar multiple session studies to look for the perspective and future direction of our findings. For instance, multiple session of tDCS in persons with mild cognitive impairment and dementia proved to significantly improve memory immediately after tDCS but not in the long run, suggesting that if used in multiple sessions, tDCS should be combined with cognitive training to reach long-term memory facilitation (see Cruz Gonzalez et al., 2018 for a review). In line with this, a recent study with healthy older adults showed that decision-making training concurrent with tDCS improves performance only in comparison with baseline, having no long-term training and transfer effects (Horne et al., 2020). Similar to the findings of Huo et al. (2018), it was shown that, also for healthy older adults, multiple tDCS sessions do not benefit executive functioning and suggest that tDCS in combination with cognitive training may be a better approach. In line with these suggestions, after pairing multiple RALC tDCS sessions with cognitive training, Nissim et al. (2019) obtained effective results, showing enhanced working memory and functional connectivity in healthy older adults. Moreover, the authors demonstrated that an increase in functional connectivity throughout multiple sessions points out that repeated stimulation may produce mechanistically different and more distributed neural effects in comparison with a single tDCS session. In line with this, a study with young adults assessed tDCS effects during multiple sessions of visuo-spatial n-back training and demonstrated significantly improved performance in the tDCS group in comparison with sham, especially in participants with low baseline performance (Katz et al., 2017). In addition, the authors show that training effects were stable in the long term after 1 year of follow-up assessment. No transfer effects were observed, however. All in all, also studies aiming at cognitive and neural augmentation due to multiple tDCS sessions show discrepancies. Although tDCS, if used with conventional protocols (i.e., less than 40 min per session; <4 mA), seems to be safe with little adverse effects during both single and multiple sessions (see Bikson et al., 2016; Nikolin et al., 2018 for a review), accumulative effects are still not clear and a possible decline in other cognitive functions but an increase in others is not well-understood (Iuculano and Kadosh, 2013). Another ethics issue arises regarding the interindividual responsiveness to tDCS, bringing benefit only for “tDCS responders” and putting “not-responders” at a disadvantage (Lavazza, 2019). Therefore, more tightly controlled, individualized, and highly powered studies in the tDCS domain are necessary to establish its neuromodulatory benefits if any.

In particular, future research with bigger sample sizes should incorporate a cluster analysis approach for behavioral and neural data while combining tDCS and fNIRS or fMRI to control for interindividual differences. Furthermore, it should be investigated how tDCS stimulation impacts on ongoing activity patterns and connectivity in frontal networks. Taking the enhancement-related null effect of bilateral tDCS into account, unilateral tDCS stimulation protocols should also be considered for future research. Previous research has shown that unilateral anodal tDCS over the right dlPFC did not affect model-based learning in a decision-making task (Smittenaar et al., 2014). Thus, unilateral anodal tDCS over the left dlPFC, a region critically involved in the learning of state transition structures in this task as well as other forms of decision-making (Wittkuhn et al., 2018; Horne et al., 2020), may be a good candidate. In addition, the modulatory efficacy of tDCS for decision-making-related learning was shown to be related to cortical thickness only in the left PFC (Filmer et al., 2019). Further research on finding efficient performance-enhancing tDCS protocols, enabling individualized and efficient interventions, would be especially beneficial for older adults showing deficits in the ability to learn sequential state structures (Eppinger et al., 2015), which nowadays gets more and more important in our everyday life.

## Conclusions

In the present study, we provide empirical evidence that fNIRS is a suitable method for measuring hemodynamic correlates of sequential decision-making. The results showed that the acquisition of sequential state transitions to predict future rewards is accompanied by enhanced dlPFC activation in younger adults. These results are consistent with a previous fMRI study using the same three-state Markov decision task in a comparable sample of young adults (Eppinger et al., 2015). Furthermore, bilateral tDCS increased hemodynamic response irrespective of reward conditions in the dlPFC, having no enhancing effect on sequential decision-making performance, whereas tDCS-induced neural upregulation seemed to be related to faster decision speed in the delayed reward condition. Taken together, the present study contributes to the further understanding of the underlying neural mechanisms of tDCS-induced modulation of cognitive functions and has significant implications for future studies with single or multiple tDCS sessions in the context of a larger body of tDCS research. Future studies with larger sample sizes, individualized stimulation protocols, more precise electrode montages, and integration of structural magnetic resonance imaging scans or 3D digitizer in fNIRS as well as short-distance channel measurement and multimodal monitoring of systemic physiology during fNIRS measurement are necessary for a deeper elaboration of interindividual differences in tDCS-induced effects on behavioral and neural correlates of sequential decision-making. Defining individual performance-enhancing stimulation protocols is especially important in light of counteracting the deteriorating cognitive functioning of a growing older population.

## Data Availability Statement

The raw data supporting the conclusions of this article will be made available by the authors upon request, without undue reservation.

## Ethics Statement

The studies involving human participants were reviewed and approved by the Ethics Committee of the Technische Universität Dresden (EK 469112015). The patients/participants provided their written informed consent to participate in this study.

## Author Contributions

SP, AD, IS, and S-CL contributed to the conception and design of the study. IS and AD organized the database, performed data collection, statistical analysis, wrote the first draft, and sections of the manuscript. All authors contributed to the revisions of the manuscript and have read and approved the submitted version.

## Conflict of Interest

The authors declare that the research was conducted in the absence of any commercial or financial relationships that could be construed as a potential conflict of interest.

## Abbreviations

BAS: behavioral activation system
BIS: behavioral inhibition system
dlPFC: dorsolateral prefrontal cortex
DPF: differential pathlength factor
fMRI: functional magnetic resonance imaging
fNIRS: functional near-infrared spectroscopy
HRF: hemodynamic response function
GLM: general linear model
ISI: interstimulus interval
ITI: intertrial interval
RT: response time
tDCS: transcranial direct current stimulation.
