# Supplementary material for: Functional Effects of Bilateral Dorsolateral Prefrontal Cortex Modulation During Sequential Decision-Making: A Functional Near-Infrared Spectroscopy Study With Offline Transcranial Direct Current Stimulation
Source: Front Hum Neurosci. 2021 Feb 3;14:605190. doi: 10.3389/fnhum.2020.605190 (PMC7886709; doi:10.3389/fnhum.2020.605190)
Supplement: Supplementary file 1 [file Data_Sheet_1.docx]

**Supplementary Materials**

Data preprocessing

In the context of the task design of the current study, the GLM modelled hemodynamic responses as a linear combination of task-related predicted responses and an error term that can be formalized as:

y(t) = β_1_x_1_(t) + β_2_x_2_(t) + β_3_x_3_(t) + β_4_x_4_(t) +*e*(t)

where y(t) is a hemodynamic state value for O_2_Hb, tHb and HHb for a given channel; x_1_(t), x_2_(t), x_3_(t) and x_4_(t) are the regressors modelling the expected signals for(i) the stimulus_(immediate)_, (ii) the outcome_(immediate)_, (iii) the stimulus_(delayed)_ and (iiii) the outcome_(delayed)_ for a given channel. The analysis will be based on the outcome regressor due to the nature of the task (see Eppinger et al., 2015). The corresponding beta values β_1_, β_2_, β_3_, β_4_are the amplitudes of the evoked hemodynamic responses; and *e* is the error term in a given channel (Barker et al., 2013; Huppert, 2016; Ye et al., 2009; Xu et al., 2014).

**Supplementary Results**

**Effects on performance**

Table S1 provides a comprehensive overview on the statistics of all effects of the applied linear mixed effects models on performance data.

***Table S1.***Statistical overview on the main and interaction effects of the linear mixed effects models with subjects as random intercepts and the factors *Treatment* (active transcranial direct current stimulation (tDCS) vs. sham-stimulation), *Condition* (i.e. immediate vs. delayed reward) and *Bin* (six learning bins per block) on response times and optimal choice performance.

| **Factor** | **Response Times** | | **Optimal Choice**  **Performance** | |
| --- | --- | --- | --- | --- |
|  | *F* statistic | *p*-value | *F* statistic | *p*-value |
| Intercept | 964.94 | <.0001 | 2392.28 | <.0001 |
| Treatment | 0.07 | 0.79 | 0.85 | 0.37 |
| Condition | 166.09 | <.0001 | 167.68 | <.0001 |
| Bin | 44.44 | <.0001 | 43.09 | <.0001 |
| Treatment × Condition | 0.18 | 0.67 | 1.90 | 0.17 |
| Treatment × Bin | 1.65 | 0.15 | 0.24 | 0.95 |
| Condition × Bin | 9.05 | <.0001 | 3.80 | .002 |
| Treatment × Condition × Bin | 33.01 | <.0001 | 0.12 | 0.99 |

# References

Barker, J. W., Aarabi, A., & Huppert, T. J. (2013). Autoregressive model based algorithm for correcting motion and serially correlated errors in fNIRS. *Biomedical Optics Express*, *4*(8), 1366–1379. https://doi.org/10.1364/BOE.4.001366

Eppinger, B., Heekeren, H. R., & Li, S.-C. (2015). Age-related prefrontal impairments implicate deficient prediction of future reward in older adults. *Neurobiology of Aging*, *36*(8), 2380–2390. https://doi.org/10.1016/j.neurobiolaging.2015.04.010

Huppert, T. J. (2016). Commentary on the statistical properties of noise and its implication on general linear models in functional near-infrared spectroscopy. *Neurophotonics*, *3*(1), 010401. https://doi.org/10.1117/1.NPh.3.1.010401

Ye, J. C., Tak, S., Jang, K. E., Jung, J., & Jang, J. (2009). NIRS-SPM: Statistical parametric mapping for near-infrared spectroscopy. *NeuroImage*, *44*(2), 428–447. https://doi.org/10.1016/j.neuroimage.2008.08.036
